# Indents

**Published:** 1921-08

**Authors:** 


### INDENTS

**☞Brief Articles of Technical or Scientific Value will receive notice and comment. Contributions invited.**

---

To Clean Trays from Modeling Compound. Soak the trays in hot water for fifteen minutes or more in concentrated ammonia and they will clean readily.—Dr. I. Margulies, in *Digest.*

---

Mouthwash. The following is a good astringent mouth wash:

| | |
|---|---|
| Rx.—Benzoic acid | gr. xv, |
| Tr. Krameria, | ʒ ss. |
| Canadian pine, | f℥ j, |
| Alcohol, | f℥ j. |

M. Sig. One teaspoonful to a glass of water.—J. A. Viverito, in *Cosmos.*

---

Toothbrush as a Cause of Mouth Infection. Cobb gives the following illustrative case: Patient, age twenty-six, suffered from repeated attacks of oral infection, always extending to throat. These attacks were followed by mild rheumatic symptoms. No evidence of dental infectious foci. Cobb advised sterilization of toothbrush by soaking in alcohol both before and after using. This proved to be a solution of the mystery.—C. M. Cobb, in *Cosmos,* July, p. 747.

---

Partial Denture vs. Removable Bridge. The following definitions are axioms which should be borne in mind when planning a case, and if the meaning of the definitions are adhered to in the construction of appliances, better results will be obtained in either branch of our science:

Any structure which through necessity *depends upon the mucous tissues and their underlying bony structure for support,* is a partial denture and not a bridge, regardless of the material of which it is constructed. It must be so designed that it can be removed at will by the patient.

Any structure which *depends upon the abutment teeth* to bear the major burden of stress of mastication, and so constructed that it can be removed at will, is a removable bridge.—A. A. Nelson, in *Summary,* p. 493.

---

NEVADA USES GAS FOR LEGAL DEATH. A recent newspaper story of the new method of capital punishment in the state of Nevada opens new fields of speculation as to the possible uses of lethal gas and its close associates in the future.

"Prior to January of this year," reads the article, "a man condemned to death in Nevada had choice of one of two ways to die. He could select the bullet or the hangman's noose. Choice was removed by the last legislature, meeting in Carson City early this year, when it passed a bill decreeing death penalties should be exacted through the use of deadly gas.

"A new tier of cells has been erected connected by invisible pipes to gigantic gas tanks. In all of the death cells, arrangements have been made so ventilation can be shut off, and in each door is a heavy glass window. In passing sentence, death is designated to take place within a week. Under Nevada's new law, when the final week arrives, any meal may be the condemned man's last meal, for in the food on the fatal day will be a strong opiate. After drowsiness has overcome the man, the gas will be released into the cell."

During the debate on the bill several legislators pointed out the fact that mental torture would not be ended with the use of lethal gas instead of the rifle or noose. All the horros of waiting must be borne under the lethal gas law, as well as under the law which necessitated choosing bullets or hanging.

Many questions concerning hunger strikes, etc., arose, but were not answered at the legislative session. The bill passed, and as yet the new method has not been tried, for no prisoners are awaiting death there at the present time.—*Current Research.*

---

ROCHESTER DENTAL DISPENSARY—STATEMENT OF WORK DONE. 1920—Tooth treatments, 50,756; Root treatments, 15,888; Root fillings, 1,682; Amalgam fillings, 10,769; Synthetic fillings, 1,082; Cement fillings, 13,255; Gutta-percha fillings, 213; Silver nitrate, 6,502; Pulps capped, 123; Crowns, 41; Inlays, 57; Orthodontia, 4,155; Extractions, 12,191; X-rays, 542; Prophylactic treatments, 782; Devitilizations, 917; Gum treatments, 578; Number of visits to dispensary, 46,911; Patients paid 5c., 41,902; Dismissals, 10,717; Prophylactic treatments in schools, 64,368; Number lectured to in schools, 46,399.

*Surgical Department*—Surgical examinations, 3,512; Tonsil and adenoid operations, 2,354.

---

THE "HARMLESS" GINGIVITIS. The initial stages of gingival infection, the precursor of the more deeply seated infections involving the peridental membrane, the alveolar structure with its cancellated spaces and myelitic contents, the alveolar periosteum and the gingival tissues, constitute a phase of the problem of dental prophylaxis, which from observation we are led to think is too often completely disregarded. Every procedure in the realm of reparative dentistry—operative and prosthetic dentistry—should be undertaken from the standpoint of the future prevention of both caries involvement and gingival irritation. The filling which improperly restores contact point, occlusal surface, embrasures, etc., prepares the field for a future pyorrheal involvement. The bridge, whether fixed or removable, which imposes upon its abutments a degree of stress greater than they may safely sustain or which subjects the occluding teeth to misdirected forces, is as successful a factor in the incidence of peridental disease as any in its varied etiology. Under these circumstances will not only be the span of life of the abutment teeth greatly shortened, but also that of every other unit of the arch, upper and lower. Any form of crown, whether of the shell or dowel variety, which impinges upon the gum tissues, are dental abominations unworthy of the aims of dentistry. No proper appreciation of the grave possibilities of the simplest form of gingival irritation can be had without a clear mental picture of the histological characteristics and relationships of the soft and calcified tissues which invest the teeth.

A marginal gingivitis may appear from a clinical standpoint as a harmless lesion devoid of serious pathologic possibilities and calling for simple therapeutic measures. It is seldom looked upon as a serious menace to not only the masticatory apparatus of the patient but also to his health. Probably as high as eighty per cent. of all men and women of the country, in middle life, are sufferers from gingival and peridental disease, and traceable in practically all instances to etiological factors centered in and around the gingival tissues. The percentage of cases of so-called systemic pyorrhea or in other words in which the disease is due to vascular disturbances manifesting themselves by changes of a retrograde nature in the alveolar bone, is so small that in the main it does not materially affect our conclusion. These cases, in the minority, have to be dealt with from an entirely different angle. Certainly four out of every five cases of involvement of the investing tissues can be definitely traced to congenital or acquired factors centered in or around the teeth. They are of local etiology, in most cases could have been prevented by the elimination of the factors responsible for the initial gingival irritation and by a proper appreciation of the physiological functions of the dental and oral tissues. As long as the tendency shall persist to institute dental reparative measures from the standpoint of individual teeth; to disregard the physiologic relations of one tooth to the other, and to practice dentistry from the standpoint of one tooth as the unit rather than the full complement of teeth as the unit, the high prevalence of dental caries and of gingival infection must persist. Dentistry will accomplish wonderful results in the line of prevention when the dental problem ceases to be a tooth problem and becomes a mouth problem.—Julio Endelman, in *Pacific Gazette.*

---

Tooth Decay Under Clasps. Dr. W. G. A. Bonwill, of Philadelphia, wrote the following in the *Items of Interest* of July, 1893: "If a clasp fits minutely all the surface of a crown, it makes of the minute space between the crown and the clasp a capillary surface, and keeps the mucous secretions, as well as the fine food, forever in contact and with no space for the circulation of the saliva. Whereas, if the band touches but a few places on the tooth crown, it will rest just as firmly if it has been well fitted in the mouth and allowed to take its own position when tried on the crown.

"Capillary power made by surfaces very closely proximated is the surest means of producing caries. Where a space is left, the points that do touch are in absolute contact, and aside from the slight wear on the tooth, the surface can not decay as when there is an actual and close fitting. If made of fine, soft gold, there would always be danger.

"A clasp is not needed to grasp the crown closely. The width of the clasp should be as great as can be made, and to steady the plate without grasping it firmly. This will be a new idea to many."

In speaking of clasped dentures in general, he further states: "I can further assure the far more perfect success of these operations if the clasps are made to touch not more than at three of four points on the crown. Where fitting accurately, caries is doubly invited by capillary action.'' Dr. Bonwill's years of experience along these lines should be worth a great deal to the profession. Study these statements, take the practical hint that is given in his message, and you will add to your own, the essence of the practical experience of the greatest authority on clasped dentures the country ever produced.—Dr. L. Underdahl, in *Northwestern Journal.*

---

TOBACCO SMOKE AS A MOUTH DISINFECTANT. Professor V. Puntoni, of the University of Rome, has undertaken some experiments with the object of ascertaining the real action of tobacco smoke as a disinfectant, under conditions similar to those which exist in the oral cavity. The results of these experiments are interesting. In the first place, it was found that the strikingly disinfectant power that tobacco smoke exercised in vitro did not occur to the same extent in the mouth of the smoker, and the most that could be said was that a bactericidal action was only shown to follow the consumption of very large quantities of tobacco, and then only on the micro-organism of least resistance, such as the meningococcus and cholera vebrio.

It is not admissible that microbes having the resistance of B. typhosus or greater can be killed in the mouth by tobacco smoke, and it is absurd to think that the bactericidal action of the smoke could manifest itself in the respiratory tract as a sequel to inhalation. The different qualities of tobacco made use of in these experiments showed a disinfecting power almost equal in relation to the weight of tobacco used; denicotinized cigars acted just as powerfully as ordinary ones. The smoke of tobacco completely decolorized by filtration through compressed cotton-wool retained a marked bactericidal action, notwithstanding the loss of all the nicotine and tar products, which are the two elements possessing definite disinfecting power. The bactericidal substances contained in this decolorized smoke are soluble in water, one of them being capable of distillation at 100 degrees F., and identical with formaldehyde, the other not capable of distillation was pyrrol, the bactericidal action of which as a component of tobacco smoke, and hitherto unknown, is important.—*The Dental Record.*

---

Extraction and Eradication of Diseased Periapical Tissue. At the present day, in the eradication of periapical infections, whether tooth extraction is part of the treatment or not, no operative work should be attempted without a preliminary radiographic examination. In these cases extraction is often only a part of the treatment, being that part which is concerned with removal of the original cause and in gaining better access to and drainage of the diseased bone area. To insure removal of the disease in the adjoining bone, extraction must be followed by curetment when the radiogram reveals the presence of a well-developed granuloma or cyst.

It should be remembered that every granuloma is a potential cyst. We almost every day have evidence of the evil results of neglect of curetting after extraction because the extractor failed to be guided by X-ray evidence. It is true that often extraction alone will enable nature to take care of the remaining periapical disease, but it is equally true that healing of a tooth socket may take place over a mass of granulation tissue or a cyst which will later give rise to serious trouble and require a second operation.

The aim of many exodontists seems to be to extract the largest number of teeth in the shortest possible time, and they are forced into this by the usual method of anesthesia with nitrous oxid. It has been said of a well-known extraction specialist that he prided himself on being able to ''keep one in the air all the time.'' Digital agility is a very desireable gift in this work, but it is often employed to the detriment of the tissues. In .the hurry incident to nitrous oxid anesthesia, teeth are often extracted with little regard for the adjoining soft tissues, the increased hemorrhage obscures the field, and there is no time to give minute attention to the diseased bone or to the proper care of the tissues in order to promote rapid and smooth healing after the extraction. These objections are all overcome by the use of novocain as a local anesthetic, which should be the anesthetic of choice in all operations for the relief of chronic periapical infections.

In curetment after extraction the operator should be guided by the extent of the disease process shown by the radiogram. The curetting need not and should not be too vigorous—just sufficient to remove or at least thoroughly loosen the diseased soft tissue without carrying infection into surrounding healthy bone. Curets of all shapes and sizes have been designed for this work, but I have found an adaptation of the ordinary chalazion curet, used by the oculist, to be most suitable. Curetment is unnecessary in cases of suppuration where no granulation tissue is present, and indeed may do harm by spreading infection.

After extraction and curetment the local anesthesia permits such treatment of the alveolar process and gums whereby a smooth healing is promoted and much time is saved before the insertion of artificial dentures. This treatment consists in trimming and smoothing of the redundant alveolar process which otherwise would require weeks and months for absorption. The free gum margins are then trimmed and approximated here and there with horse-hair sutures. These sutures are so placed that they will permit the escape of any secretion that may accumulate from the infected tooth sockets.—Robert H. Ivy, in *Cosmos*, Page 574.

